# Identification of gut bacteria reductases that biotransform steroid hormones

**DOI:** 10.1038/s41467-025-61425-6

**Published:** 2025-07-08

**Authors:** Gabriela Arp, Angela K. Jiang, Keith Dufault-Thompson, Sophia Levy, Aoshu Zhong, Jyotsna Talreja Wassan, Maggie R. Grant, Yue Li, Brantley Hall, Xiaofang Jiang

**Affiliations:** 1 Department of Cell Biology and Molecular Genetics, University of Maryland, College Park, College Park, MD USA; 2 National Library of Medicine, National Institutes of Health, Bethesda, MD USA; 3 Division of Molecular and Cellular Biology, Eunice Kennedy Shriver National Institute of Child Health and Human Development, National Institutes of Health, Bethesda, MD USA; 4 Department of Computer Science, Maitreyi College University of Delhi, Delhi, India; 5 Department of Chemistry and Biochemistry, University of Maryland, College Park, College Park, MD USA; 6 Center for Bioinformatics and Computational Biology, University of Maryland, College Park, College Park, MD USA

**Keywords:** Microbial genetics, Enzymes

## Abstract

The metabolism of steroid hormones by the gut microbiome is increasingly recognized as a key factor in human health; however, the specific enzymes mediating these transformations remain largely unidentified. In this study, we identify Δ^4^-3-ketosteroid 5β-reductase, 3β-hydroxysteroid dehydrogenase/Δ^5-4^ isomerase, and Δ^6^-3-ketosteroid reductase enzyme families encoded by common human gut bacteria. Through phylogenetic reconstruction and mutagenesis, we show that 5β-reductase evolved to specialize in converting both natural and synthetic 3-ketosteroid hormones into their 5β-reduced derivatives, while Δ^6^-3-ketosteroid reductase adapted to produce Δ^6^-reduced derivatives. We also find that the novel 3β-hydroxysteroid dehydrogenase/Δ^5-4^ isomerase is fused with 5β-reductase in multiple species, streamlining the conversion of pregnenolone, a 3β-hydroxy-5-ene and steroid hormone precursor, into epipregnanolone. Through metagenomic analysis, we reveal that these enzymes are prevalent in healthy populations and enriched in females compared to males. These findings lay the groundwork for mechanistic investigations into how microbial steroid metabolism modulates host hormonal physiology.

## Abstract (web summary)

Here, the authors identify and characterize three gut bacterial enzyme families that metabolize endogenous and synthetic steroid hormones, showing to act on diverse substrates, to exhibit gene fusions, and gender-linked prevalence, opening avenues for studying mechanisms of microbial-mediated steroid metabolism and host hormonal physiology.

## Introduction

The human gut is increasingly recognized as a major site of steroid metabolism, where microbial communities transform cholesterol, bile acids, and steroid hormones into various metabolites. Over the past decade, researchers have identified several gut microbial enzymes involved in these transformations, including cholesterol dehydrogenase (*ismA*)^1^, steroid sulfotransferase (*BtSULT*)^2^, bile acid hydrolase (*BSH*)^3^, the bile acid-inducible (*bai*) operon^4^, and steroid-17,20-desmolase (*desAB*)^5^. These enzymatic discoveries have enhanced our understanding of how the microbiome contributes to steroid homeostasis and revealed potential connections between microbial steroid metabolism and diverse health outcomes, including cardiovascular disease^6^, pathogen resistance^7^, and prostate cancer^8^.

Among these compounds, steroid hormones, including progestogens, estrogens, androgens, glucocorticoids, and mineralocorticoids, are cholesterol-derived signaling molecules whose precise physiological balance is critical for the regulation of reproduction, metabolism, stress response, and immunity^9–13^. Steroid hormones and their synthetic analogs are widely used as pharmaceuticals. Regardless of whether they are endogenously produced or orally administered, these compounds frequently reach the gut via biliary excretion or direct ingestion. Endogenous steroid hormones are secreted into bile and enter the gut in substantial quantities; for example, a study has shown that pregnant women can eliminate up to 33 mg of these hormones daily through feces^14^. Similarly, exogenous steroid hormones reach the gut in physiologically significant amounts. In a bile fistula rat study, ~70% of injected [4-¹⁴C]pregnenolone was recovered in bile within 24 h, primarily as pregnenolone sulfate^15^.

A significant fraction of unsaturated steroid hormones reaches the intestinal tract, providing a rich source of substrates for microbial metabolism. While most steroids excreted via bile are conjugated and saturated, certain 3β-hydroxy-5-ene steroids—including pregnenolone^16,17^, dehydroepiandrosterone (DHEA)^18,19^, androstenediol^20^, and 5-pregnene-3β,20α-diol^16,17^—maintain their unsaturated structure during excretion, predominantly existing as sulfated derivatives without undergoing A-ring saturation. An illustrative example is DHEA sulfate (DHEAS), the most abundant circulating steroid sulfate in humans, with adult serum concentrations typically ranging from 1 µmol/L to 10 µmol/L^21^. Studies have demonstrated that in rat models, ~29.1% of intravenously administered DHEAS is excreted unchanged into the bile^22^. Similarly, while unconjugated progesterone represents a minor fraction of total excreted hormone, its detection- 1.5% of an injected dose recovered unmetabolized in bile^23^—underscores a potentially important pool available for microbial transformation and downstream metabolic activity. Collectively, these unsaturated steroids, in both conjugated and free forms, represent a biologically significant reservoir of substrates available for microbial biotransformation in the gut.

Products resulting from microbial steroid transformations can either be excreted in feces or reabsorbed into the circulation, influencing host-hormone signaling and systemic physiology^24^. A compelling example involves the gut microbial conversion of cortisol into 11β-hydroxyandrostenedione, a metabolite lacking the C-21 side chain. This metabolite circulates and effectively inhibits renal 11β-hydroxysteroid dehydrogenase type 2, causing cortisol-mediated activation of mineralocorticoid receptors, sodium retention, and hypertension. This mechanistic link is underscored by experiments demonstrating that microbiome transfers from hypertensive human donors into germ-free mice induce hypertension, establishing causality^25^. Additionally, specific bacterial consortia can convert glucocorticoids to 17α-hydroxy-progestins (progestogens) under anaerobic conditions involving hydrogen gas. For instance, co-culturing *Gordonibacter pamelaeae* with hydrogen-producing *E. coli* yields microbial progestins chemically identical to human progesterone receptor ligands, which have been shown to be absorbed in the colon and enter systemic circulation^26^. Moreover, the gut bacterium *Clostridium innocuum* significantly influences host endocrine function by converting progesterone to epipregnanolone, a neurosteroid with substantially reduced progestogenic activity^27^. Colonization by *C. innocuum* in mouse models decreases circulating progesterone levels by 35%, halts ovarian follicular development, and disrupts estrous cycling^28^. These observations highlight a crucial bidirectional relationship in which host-derived steroids undergo microbial biotransformation within the gut, subsequently influencing the human endocrine system.

Microbial reduction of steroid hormones, such as progesterone, into stereospecific 3α,5β- and 3β,5β-derivatives was first described in the early 1960s using *Clostridium* species^29^. Subsequent studies have confirmed these reactions in species such as *Clostridium innocuum* and *Clostridium paraputrificum*, which rapidly convert progesterone into 5β-reduced metabolites under anaerobic conditions^30,31^. These transformations primarily yield dihydro and tetrahydro derivatives—most notably, the 3α,5β isomer—that exhibit significantly reduced progestational activities. Microbial inactivation of progesterone has been proposed to explain its low oral bioavailability and diminished therapeutic efficacy^32^. Stokes and Hylemon (1985) further characterized these microbial conversions by identifying the reductase activities of *C. innocuum*, directly connecting microbial metabolism to the generation of neuroactive steroid metabolites^30,33^. Despite these advances, the specific genes encoding these steroid-transforming enzymes remained unknown, limiting our mechanistic understanding of progesterone metabolism by gut bacteria and its broader physiological consequences—a gap our current study seeks to bridge.

In this study, we characterized three microbial enzyme families responsible for steroid hormone metabolism in the mammalian gut. We first identified a Δ^4^-3-ketosteroid 5β-reductase enzyme family that catalyzes the reduction of progesterone to 5β-dihydroprogesterone, filling a knowledge gap in this pathway in the gut microbiome. We found that this 5β-reductase can act on a variety of steroid hormones, including cortisone and multiple progestins, and identified evolutionary changes in key residues that support the specialized metabolism of steroid hormones. In addition, we identified a novel 3β-hydroxysteroid dehydrogenase/Δ^5-4^ isomerase that converts pregnenolone to progesterone and transforms 5β-dihydroprogesterone into epipregnanolone. Interestingly, this gene often fuses naturally with 5β-reductase, thereby streamlining the entire pathway from 3β-hydroxy-5-ene steroids to 3β-hydroxy-5β-reduced steroids. We also characterized a Δ^6^-3-ketosteroid reductase enzyme family that reduces 6-dehydroprogesterone to progesterone. We performed a metagenomic survey on 1549 samples and observed that 5β-reductase and 3β-hydroxysteroid dehydrogenase/Δ^5-4^ isomerase, while relatively prevalent in the general population, are more prevalent and abundant in females. The three enzyme families we identified and characterized in this study highlight the diverse mechanisms through which gut bacteria have evolved to metabolize host steroids, providing a molecular basis for understanding how the gut microbiota may interact with host hormones and impact host health.

## Results

### Identification of 5β-reductase *ci2350* in *Clostridium innocuum*

**Fig. 1:**
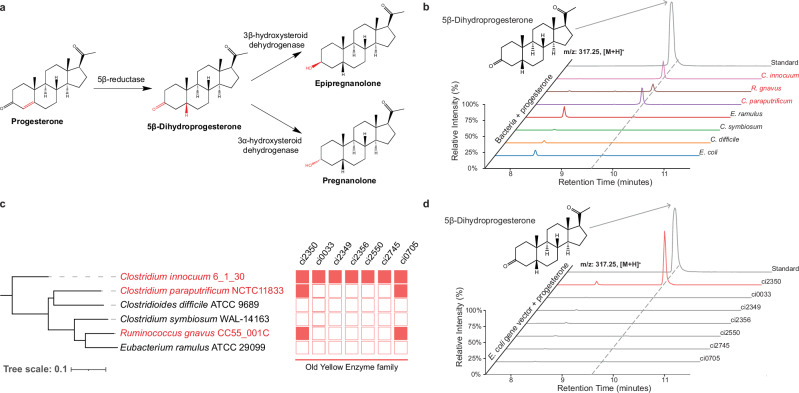
Identification of the progesterone 5β-reductase gene family. **a** Pathway for the conversion of progesterone to pregnanolone or epipregnanolone. **b** Detection of 5β-dihydroprogesterone in cultures of seven bacterial strains incubated with progesterone. **c** Phylogenetic tree and heatmap illustrating the distribution of Old Yellow Enzyme family genes across various bacterial strains. The red-highlighted strains (*Clostridium innocuum* 6_1_30, *Clostridium paraputrificum* NCTC11833, and *Ruminococcus gnavus* CC55_001C*)* indicate strains with detected enzymatic activity of 5β-reductase. In the heatmap, filled red boxes represent the presence of the corresponding gene, whereas empty red boxes represent the absence of the corresponding gene. **d** Comparative chromatograms showing the presence of 5β-dihydroprogesterone in the cultures of *ci2350* transformed *E. coli* with the strongest activity (highlighted in red) compared to other Old Yellow Enzyme-transformed *E. coli*. The chromatograms demonstrated distinct peaks corresponding to 5β-dihydroprogesterone, confirming the enzymatic activity of *ci2350*.

The proposed progesterone reduction pathway consists of a 5β-reductase enzyme that catalyzes the reduction of progesterone to 5β-dihydroprogesterone, and two additional enzymes, 3α-HSDH (3α-hydroxysteroid dehydrogenase) and 3β-HSDH (3β-hydroxysteroid dehydrogenase) that catalyze the conversion of 5β-dihydroprogesterone to pregnanolone or epipregnanolone, respectively (Fig. 1a)^33^. To identify a putative progesterone 5β-reductase enzyme, we used metabolomics to assay for the reduction of progesterone in cultures of potential progesterone reducers and non-reducers, and comparative genomics to identify putative reductase genes. We confirmed that *Clostridium innocuum* 6_1_30 *and Clostridium paraputrificum* NCTC11833 were able to reduce progesterone, corroborating previous findings^30,33^ (Fig. 1b). *Ruminococcus gnavus* (*Mediterraneibacter gnavus)* C55_001C, a common microbe in the human gut, also reduces progesterone levels, demonstrating a previously unknown function of this bacterium associated with various facets of human health and disease^34^ (Fig. 1b). We did not observe any reduction of progesterone in the cultures of *Eubacterium ramulus* ATCC29099, *Clostridium symbiosum* WAL-14163, *Clostridium difficile* ATCC9689, or *Escherichia coli* IM93B (Fig. 1b).

We reasoned that the putative progesterone 5β-reductase genes should be present in the progesterone-reducing species and absent in the species that did not reduce progesterone. Previous characterization of Δ^4-3^-ketosteroid 5β-reductases from *C. innocuum* and *C. paraputrificum* has suggested that these enzymes utilize NADH/NADPH, FMN, and likely FAD as cofactors, and they likely contain iron-sulfur (Fe-S) clusters^33^. Combined with the fact that the 5β-reductase is proposed to be an ene-reductase reaction (Fig. 1a), we hypothesized that 5β-reductase would be part of the Old Yellow Enzyme family (COG1902), a family of enzymes that often catalyzes stereospecific reduction reactions and is involved in many biologically and medically significant reactions^35^. We identified seven putative Old Yellow Enzymes in the *C. innocuum* genome, two of which had orthologs in *C. paraputrificum* and *R. gnavus* and were absent in the progesterone non-reducing species (Fig. 1c). To determine whether any of these enzymes had 5β-reductase activity, we transformed *E. coli* strains with each of the *C. innocuum* Old Yellow Enzyme orthologs and tested them for progesterone reduction (Fig. 1d). Two of the genes, *ci2350* and *ci0705*, matched the expected presence and absence patterns (Fig. 1c), but only *ci2350* showed detectable 5β-reductase activity, confirming that *ci2350* encodes a 5β-reductase. Consistent with reductive ene reactions observed in other Old Yellow Enzymes, the reduction of the steroid double bond by ci2350 is coupled to NADH oxidation, with progesterone acting as the terminal electron acceptor—a reaction characteristic of anaerobic, reducing conditions in the gut (Supplementary Fig. 2).

### *ci2350* reduces both natural and synthetic steroid hormones

**Fig. 2:**
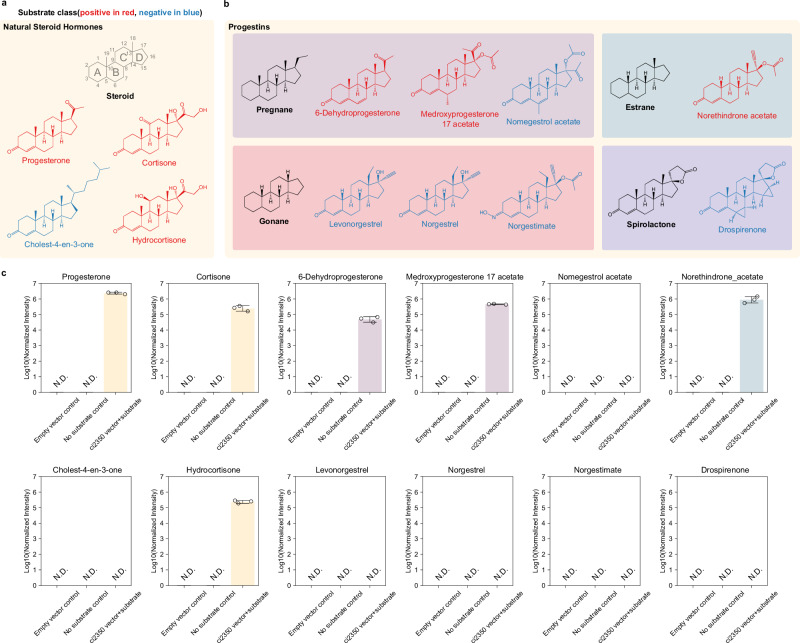
Selective reduction of steroids by progesterone 5β-reductase. **a** Structure of natural steroid hormones. **b** Synthetic progestin structures were arranged into categories based on their structures and precursors. The steroid core structure with labeled rings is shown in panel a. Backbone structures for the different types of metabolites are shown in black in each box. Metabolites colored in red were reduced by *ci2350*, whereas metabolites shown in blue were not reduced. **c** Bar graphs showing mass spectrometry-quantified results normalized by intensity from biological triplicates of substrates incubated with cultures of *ci2350* transformed *E. coli*. These were compared to substrates incubated with *E. coli* transformed with the vector backbone control and media control. Colors of bars in reduced metabolites reflect the grouping present in (**b**) and data are presented as mean values +/- SEM.

We investigated the activity of the *C. innocuum* 5β-reductase on various steroids. While progesterone is a probable natural substrate for this enzyme, several metabolites exhibiting analogous structural features and bonding patterns may also serve as potential substrates for 5β-reductase. Using *E. coli* transformed with the *C. innocuum* gene *ci2350*, we tested for the reduction of progesterone and eleven metabolites that have the same C4 to C5 carbon-carbon double bond on the A-ring of the steroid core as progesterone, including three naturally occurring hormones (Fig. 2a) and eight synthetic progestins (Fig. 2b). All steroid biotransformation assays were conducted using standardized substrate concentrations (1 mg/100 mL) and fixed incubation times (48 h), allowing consistent and reproducible comparisons of enzyme activity across different substrates and strains. Among the natural steroids, we observed reduction in progesterone, cortisone, and hydrocortisone (Fig. 2c). In contrast, we did not observe any reduction of cholest-4-en-3-one (Fig. 2a). Cholest-4-en-3-one has a much longer hydrocarbon tail and different stereochemistry on the D-ring of the steroid core, potentially preventing the enzyme from reducing this metabolite. This corroborates previous studies where enzyme extracts from *C. innocuum* were able to reduce progesterone and testosterone, but not able to reduce cholest-4-en-3-one^33^. A related study also demonstrated that these enzymes have broad activity on steroid hormones, including corticosterone, prednisolone, and testosterone^36^.

To test if the 5β-reductase can reduce synthetic progestins, we assayed for the reduction of eight progestins derived from four different precursors. Progestin compounds, synthetic progesterone analogs that are often used in birth control and hormonal therapies^9,10^, can be generally grouped into categories based on what precursors they are synthesized from (*e.g*., progesterone, testosterone, and 19-norprogesterone) and their activity in the body. When *E. coli* transformed with *ci2350* was incubated with each progestin, we only observed reduction of the progestins in the pregnane and estrane groups, while the gonanes and spironolactone derivatives were not reduced (Fig. 2c). These differences in reactivity are likely the result of variations in the structure of the metabolites. The metabolites that are reduced have methyl groups at the C18 and C19 positions, a few modifications to the rest of the steroid ring core, and an unmodified carbonyl group at the C3 carbon (Fig. 2a, b). The exception to this trend is seen in the estrane norethindrone acetate, which is not methylated at the C19 position. The remaining metabolites exhibited various structural differences. The gonane progestins lack methyl groups at the C19 position and have an ethyl group instead of a methyl group at the C18 position. Nomegestrol acetate has an additional α, β, *γ*, *δ*-unsaturated ketone structure and an additional methyl group on the B ring of the steroid core. Norgestimate does not have the typical electron-withdrawing carbonyl group at the C3 carbon, and drospirenone has two cyclopropanes and a *γ*-lactone modification on the steroid ring structure (Fig. 2b). We also assayed for the reduction of medroxyprogesterone 17 acetate and norethindrone acetate by *C. innocuum and* found that the strain was able to catalyze near complete reduction of both progestins (Supplementary Fig. 3). These variations in metabolite structure likely contribute to the differences in the biochemical properties of the metabolites, which may explain the differences in 5β-reductase activity.

To further support the involvement of ci2350 in reducing these metabolites, we used isothermal titration calorimetry (ITC) to assess binding interactions. Enzyme expression was confirmed by SDS-PAGE prior to both transformation and ITC assays to verify production of the recombinant protein. The isolated and purified ci2350 protein was titrated with four steroid substrates: cortisone, progesterone, hydrocortisone, and 6-dehydroprogesterone, which were previously shown to be reduced in our assays (Supplementary Fig. 4). All substrates showed binding to recombinant ci2350, with dissociation constants (Kd) in the submicromolar range. Progesterone exhibited the strongest binding affinity (Kd = 4.32 × 10⁻⁷ M), followed by 6-dehydroprogesterone (Kd = 5.94 × 10⁻⁷ M), hydrocortisone (Kd = 6.68 × 10⁻⁷ M), and cortisone (Kd = 7.59 × 10⁻⁷ M). The binding was exothermic for all substrates (ΔH: –192 to –241 kJ/mol). Using the independent binding model, the stoichiometry values for all tested steroids were close to 1, indicating that one ligand bound to one ci2350 enzyme at a single binding site. These results show that the ci2350 enzyme preferentially binds progesterone-like steroids, with slight differences in affinity and thermodynamic profiles among substrates.

All the natural hormones and synthetic progestins tested have a variety of effects on the human body through their activity as agonists and antagonists of different receptors^37^. Synthetic progestins are used as therapeutics for fertility and menstrual disorders (*e.g*., 6-dehydroprogesterone), as oral contraceptives (*e.g*., medroxyprogesterone 17 acetate, norethindrone acetate), and as emergency contraceptives (*e.g*., levonorgestrel). Notably, medroxyprogesterone 17 acetate was also effective in treating metastatic breast cancer^38^. With a clearer understanding of the genes and microbial species involved in metabolizing these steroid compounds, we can better understand how their bioavailability and physiological effects are altered.

### Natural fusion enzyme converts pregnenolone to epipregnanolone

**Fig. 3:**
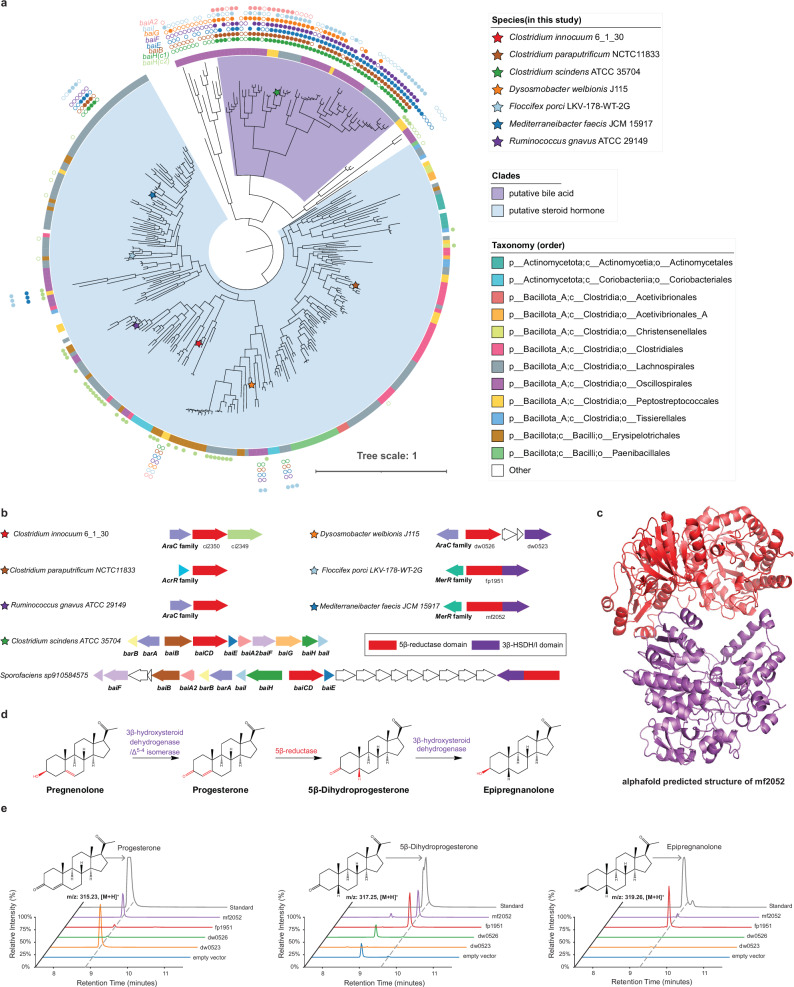
Detection of fused genes composed of 3β-HSDH/Δ^5-4^ isomerase and 5β-reductase. **a** Phylogenetic tree of putative steroid 5β-reductase clades rooted by an outgroup. The outer ring indicates the presence of the *bai* operon genes in the same genome; filled circles represent genes in close proximity, while unfilled circles represent genes in the same genome but not in close proximity. The inner ring shows the taxonomic order. Species included in this study are marked with colored stars. **b** Gene clusters associated with steroid 5β-reductase in various bacterial strains. Arrows represent different genes with colors corresponding to specific gene functions. **c** AlphaFold-predicted structure of the fused gene *mf2052* composed of 3β-HSD/Δ^5-4^ isomerase and 5β-reductase, showing the structural domains responsible for each enzymatic activity. **d** The proposed pathway for the conversion of pregnenolone to epipregnanolone, which could be mediated by different domains of the fused enzyme; **e** Chromatograms showing the detection of steroid metabolites produced by *E. coli* expressing the genes when using pregnenolone as the substrate.

Using the *C. innocuum* 5β-reductase ci2350 protein sequence as a query, we identified a putative 5β-reductase clade containing 318 genes from 282 species, primarily from the Bacillota_A and Bacillota phyla (Fig. 3a, Supplementary Data 1). This clade includes several microbial taxa commonly found in the healthy human gut, including members of the Clostridiaceae and Ruminococcaceae families. This suggests that this function may be relatively common in the human gut. The 5β-reductase homologs are frequently adjacent to transcription regulators, which vary across the AraC, AcrR, and MerR families (Fig. 3b). These homologs are often found next to other enzyme-encoding genes, including BaiH homologs and those containing a Rossmann-fold (SSF51735) as a separate gene (Fig. 3b). Interestingly, the Rossmann-fold (SSF51735)-containing gene was fused directly to the 5β-reductase gene at the C-terminus in 61 of the 257 homologs (Fig. 3b, c). The Rossmann-fold domains of the three genes share remote homology with PF01073, 3β-hydroxysteroid dehydrogenase/Δ^5-4^ isomerase (3β-HSDH/I), which is involved in steroid hormone biosynthesis in eukaryotes. In humans, the conversion of pregnenolone, the precursor metabolite of steroid hormones^39^, to progesterone is catalyzed by 3β-hydroxysteroid dehydrogenase/Δ^5-4^ isomerase^40^. Therefore, we suspected that these bacterial 3β-HSDH/I domain-containing genes were involved in the metabolism of pregnenolone and, potentially, 5β-dihydroprogesterone. Given that fused genes are frequently involved in pathway integration^41^, we hypothesized that the 3β-HSDH/I domain may provide these enzymes with the ability to catalyze the conversion of pregnenolone to progesterone, 5β-dihydroprogesterone to epipregnanolone, or both, in addition to the conversion of progesterone to 5β-dihydroprogesterone, which is catalyzed by the 5β-reductase domain (Fig. 3d).

To determine whether the fused genes perform additional 3β-hydroxysteroid dehydrogenase/Δ^5-4^ isomerase functions, we cloned representative fused genes from *Mediterraneibacter faecis* JMC15917 (*mf2052*) and *Floccifex porci* LKV-178-WT-2G (*fp1951*), a 5β-reductase gene from *Dysosmobacter welbionis* J116 (*dw0526*), and separated putative 3β-HSDH/I genes from *D. welbionis J115* (*dw0523*) into *E. coli* and assayed for the conversion of pregnenolone to the potential downstream metabolites progesterone, 5β-dihydroprogesterone, and epipregnanolone. The fused genes (*fp1951* and *mf2052*) and the *D. welbionis* 3β-HSDH/I gene (*dw0523*) could metabolize pregnenolone, with *dw0523* producing only progesterone and the fused genes producing a mixture of progesterone, 5β-dihydroprogesterone, and epipregnanolone (Fig. 3e). The *D. welbionis* 5β-reductase (*dw0526*) gene alone did not show any activity on pregnenolone, suggesting that pregnenolone conversion to progesterone is catalyzed by the 3β-HSDH/I domain (Fig. 3e). Together, these results demonstrate the functionally distinct routes for pregnenolone metabolism in the gut microbiome, with some bacteria encoding multi-enzyme pathways and others having streamlined pathways catalyzed by fused multi-functional reductases.

We aimed to determine whether the capacity to convert pregnenolone to progesterone is exclusive to the 3β-hydroxysteroid dehydrogenase/Δ^5-4^ isomerase (3β-HSDH/I) family identified in this study or is also present in previously recognized 3β-HSDH enzymes. The 3β-HSDH enzyme from *R. gnavus* catalyzes a 3β-dehydrogenation reaction on bile acids^42^, which is akin to the conversion of 5β-dihydroprogesterone to epipregnanolone. Close homologs of 3β-HSDH identified in *C. innocuum* 6_1_30 may explain the phenotype of *C. innocuum* that converts progesterone to epipregnanolone. To investigate the functions of these genes, we compared the activity of 3β-HSDH genes from *R. gnavus* C55_001C and *C. innocuum* 6_1_30 to that of *D. welbionis* 3β-HSHD/I (Supplementary Fig. 5). The *D. welbionis* 3β-HSDH/I (*dw0523*), the *R. gnavus* 3β-HSDH, and the *C. innocuum* 3β-HSDH genes were all able to convert 5β-dihydroprogesterone to epipregnanolone, demonstrating that these genes have the same 3β-dehydrogenase function, but only the *D. welbionis* 3β-HSHD/I (*dw0523*) can convert pregnenolone to progesterone (Supplementary Fig. 5, Supplementary Fig. 6). Since multiple stereoisomers of the 5β-reduced products may exist, we tested for the ability of the *D. welbionis* 3β-HSDH/I to convert 5β-dihydroprogesterone into potential terminal 3β or 3α reduced products. We found that the enzyme produced epipregnanolone (3β,5β) (Supplementary Fig. 7). This demonstrates that the 3β-hydroxysteroid dehydrogenase/Δ^5-4^ isomerase function is unique and not observed in other bacterial 3β-HSDH enzymes. To the best of our knowledge, this is the first documented instance of a bacterial enzyme exhibiting 3β-HSDH/I function on steroid hormones, revealing a novel pathway in microbial steroid metabolism.

Pregnenolone is a key precursor of other steroid hormones and altered pregnenolone levels have been implicated in various psychiatric disorders, including schizophrenia and bipolar disorder^43^. The sulfated derivatives of pregnenolone, such as pregnenolone sulfate, are known to undergo biliary excretion, thus reaching the gut. Overall, the characterization of these bacterial enzymes demonstrated the presence of multiple steroid hormone reduction pathways. Specifically, the streamlined conversion of 3β-hydroxy-5-ene steroids into 3β-hydroxy-5β-reduced steroids via the activity of a fused gene may enable these microbes to efficiently metabolize host-derived steroids, potentially providing them with a competitive advantage over other gut microbial species.

### Phylogenetic characterization of steroid 5β-reductase

**Fig. 4:**
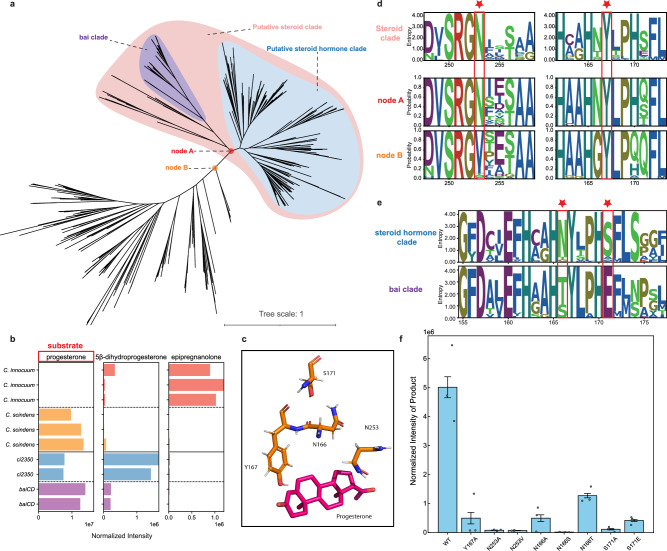
Delineation and subfunctionalization of steroid 5β-reductase. **a** Phylogenetic tree of steroid 5β-reductase related genes, highlighting the bile acid clade (purple), putative steroid clade (pink), and putative steroid hormone clade (blue). Nodes A and B are marked, representing key divergence points in the tree. **b** Bar plots showing the detection of steroid metabolites produced by *Clostridium innocuum 6_1_30* (red), *Clostridium scindens ATCC 35704* (orange), and *Escherichia coli* transformed with *baiCD* (purple) and *ci2350* (blue). Progesterone was used as the substrate (highlighted in the red box). Each bar panel corresponds to the total ion count of the metabolites (progesterone, 5β-dihydroprogesterone, and epipregnanolone) detected by LC-MS. Each species results are reported in triplicates and heterologously expressed genes are reported in duplicates. **c** 3D structure of the active site of the enzyme with the substrate bound by progesterone, highlighting the key residues involved in substrate binding and catalysis. **d** Sequence logos for catalytic residues in the Steroid 5β-Reductase clade and the ancestral state reconstructed probability of the putative steroid 5β-reductase (node A) and the outgroup (node B) (highlighted with red stars). **e** Sequence logos for key differential residues between the progesterone and bile acid clades (highlighted with red stars). **f** Bar graph shows the normalized intensity of 5β-dihydroprogesterone detected in the identified enzymes across different mutations, indicating the product abundance. Data are presented as mean values +/- SEM.

Based on the genomic context and phylogenetic relationships, we grouped the *ci2350* homologs into a putative steroid hormone 5β-reductase clade and a *baiCD* bile acid reductase clade (Fig. 3a, Fig. 4a). The *baiCD* genes encode enzymes that catalyze the reduction of 3-oxo-Δ⁴ bile acids and is a part of the relatively conserved *bai* operon^4^. Fifteen genomes encode genes from both the *baiCD* bile acid reductase clade and the steroid hormone reductases clade. For example, in *Sporofacians sp910584575*, a steroid 5β-reductase gene and a *bai* operon are in proximity, suggesting that there may be functional differences between *baiCD* and the putative steroid hormone 5β-reductase clade (Fig. 2b). In total, the differences in genomic context and the presence of both genes in some genomes suggest that the steroid hormone 5β-reductase clade is distinct from the *baiCD* enzyme family.

Next, we sought to determine if there was a functional overlap between the two clades by testing the ability of *C. innocuum* and *C. scindens* to metabolize progesterone. Previous studies have shown that *C. scindens*, although well-known for its bile acid 7α-dehydroxylation activity, did not reduce progesterone^44^. Cultures of *C. innocuum* were able to convert progesterone into a mixture of nearly all epipregnanolone, with a small amount of detectable 5β-dihydroprogesterone remaining (Fig. 4b). In contrast, the *C. scindens* cultures showed very little activity, with only trace amounts of both potential products detected (Fig. 4b). Cloning of the two genes, *C. innocuum ci2350* and *C. scindens baiCD*, into *E. coli* confirmed that these gene products were responsible for the reductase activity on progesterone, and showed that *baiCD* had limited, but detectable, reduction of progesterone (Fig. 4b). While the *C. scindens baiCD* gene may still be capable of catalyzing steroid hormone reduction, differences in cellular transport and regulation may account for the lack of activity seen in the *C. scindens* cultures (Fig. 4b). Based on these results, we hypothesized that the enzymes may have some substrate promiscuity but are from distinct enzyme clades specializing in the metabolism of bile acids or steroid hormones.

To investigate the key difference between the steroid 5β-reductase and bile acid reductase clades, we first aligned the predicted ci2350 to the previously characterized 2,4-dienoyl CoA reductase structure (PDB: 1PS9) and identified plausible regions in ci2350 that could interact with a substrate based on what is known about the active sites of the other enzymes^45^ (Fig. 4e, Supplementary Fig. 8). Based on this, we identified multiple residues in the active site of the enzyme that could potentially interact with progesterone (Fig. 4e). Positions Y167 structurally align with a key catalytic residue from the 1PS9 structure (Y166)^45^, whereas the N166, S171, and N253 positions are predicted to be in the region of the 5β-reductase enzyme active site (Supplementary Fig. 9). We then used this structural prediction to compare the sequence and functional differences between the steroid hormone 5β-reductase family and the bile acid reductase family.

With a putative steroid hormone 5β-reductase family defined, we then identified key differences between the putative bile acid reductases and steroid hormone reductases that contribute to their differences in function. When examining the conservation of the putative active site residues in the steroid hormone reductases, we found that the bile acid reductase family differed from the steroid hormone reductase family at two potentially interacting positions within the active site: N166, which is primarily tyrosine or serine in the bile acid reductases, and S171, which is primarily a glutamic acid in the bile acid reductases (Fig. 4d). Mutants of ci2350 with position N166 mutated to a neutral amino acid with no side chain (N166A) or to serine (N166S), an amino acid with a smaller side chain, showed a nearly complete loss of reductase activity, while mutation to tyrosine (N166T), the most common amino acid at this position in the bile acid reductase family, resulted in detectable but reduced activity compared to the wild type (Fig. 4f). Similarly, mutation of position S171 to an alanine (S171A), a residue with no side chain, or to the most common residue in the bile acid clade (S171E), decreased the progesterone reductase activity of ci2350 to low but detectable levels. The retention of partial activity in mutants reflecting the common residues seen in the bile acid clade highlights the partial redundancy observed when testing the activity of the *C. innocuum* and *C. scindens* proteins. These patterns demonstrate the distinct evolutionary trajectories of these enzyme clades and provide valuable insights into the complex evolutionary history of steroid metabolism in bacteria.

### Δ^6^-3-ketosteroid reductase converts steroids to Δ^6^-reduced forms

**Fig. 5:**
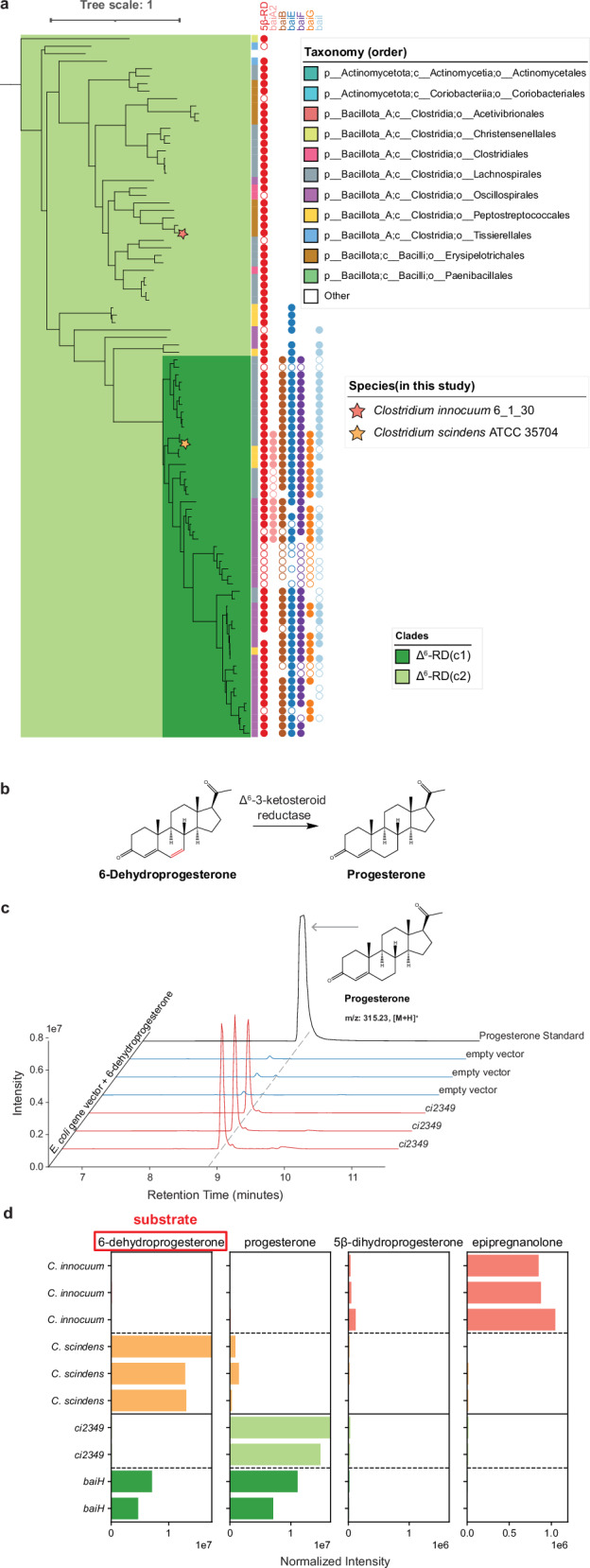
Δ^6^-3-ketosteroid reductase phylogeny and activity in 6-dehydroprogesterone reduction. **a** Phylogenetic tree of *baiH* homologs, highlighting clades *baiH*(c1) and *baiH*(c2). The circles next to the leaf indicate the presence of the *bai* operon genes in the same genome, with filled circles representing genes in close proximity and unfilled circles representing genes in the same genome, but not in close proximity. The bar next to the leaf indicates the taxonomic order of species. Species included in this study are marked with stars: *Clostridium innocuum* 6_1_30 (red star) and *Clostridium scindens* ATCC 35704 (blue star). **b** Proposed reaction for Δ^6^-3-ketosteroid reductase. **c** Chromatograms showing that *E. coli* transformed with *ci2349* converts 6-dehydroprogesterone to progesterone. **d** Bar plots showing the detection of steroid metabolites produced by *Clostridium innocuum* 6_1_30 (orange), *Clostridium scindens* ATCC 35704 (green), and *E. coli* transformed with *baiH* (dark green) and *ci2349* (light green). The substrate used is 6-dehydroprogesterone (highlighted in red). Each bar panel corresponds to the total ion count of metabolites (6-dehydroprogesterone, progesterone, 5β-dihydroprogesterone, and epipregnanolone) detected using LC-MS. Each species results are reported in triplicates and heterologously expressed genes are reported in duplicates.

In the *C. innocuum* genome, the 5β-reductase gene (*ci2350*) is adjacent to *ci2349*, which is a homolog of *baiH* (Fig. 3b), the Δ^6^-3-ketosteroid reductases that are known to be involved in bacterial bile acid metabolism^46^. When we performed a broader search for genes similar to *ci2349*, we identified 94 putative genes. Of the 94 genes identified, 92 are co-present with the 5β-reductase gene in the genome, with 78 being located directly adjacent to it (Fig. 5a, Supplementary Data 2). The 94 Δ^6^-3-ketosteroid reductase (Δ^6^-RD) genes can be categorized into two clades. The first, Δ^6^-RD(c1), comprises 51 genes that are closely related to the *baiH* gene clade. These genes not only co-localize with the previously identified bile acid reductase clade within the steroid reductase family (Fig. 4a) but are also predominantly associated with the traditionally defined *bai* operon^47^. The second clade, Δ^6^-RD(c2), is likely linked to steroid hormone reductases, suggesting specialized functions that may parallel those of the steroid hormone 5β-reductase.

To test the function of Δ^6^-RD(c2), we assayed for the reduction of 6-dehydroprogesterone, a synthetic progestin, to progesterone. The conversion of 6-dehydroprogesterone to progesterone consists of the reduction of one of the two carbon-carbon double bonds present on the B ring of the steroid nucleus, which is the same type of reduction catalyzed by BaiH enzymes^48^ (Supplementary Fig. 9), suggesting that this is a possible function of the Δ^6^-RD(c2) enzymes (Fig. 5b). To test the function of *ci2349* we tested the ability of a transformed *E. coli* strain with *ci2349* finding that it was able to convert 6-dehydroprogesterone into progesterone (Fig. 5c). When incubated with 6-dehydroprogesterone, *C. innocuum* showed nearly complete conversion of the 6-dehydroprogesterone to epipregnanolone while *C. scindens* showed only low amounts of conversion to progesterone (Fig. 5d). When the *baiH* gene from *C. scindens* and the Δ^6^-RD homolog from *C. innocuum* (*ci2349*) were transformed into *E. coli*, we observed efficient conversion of 6-dehydroprogesterone to progesterone by *ci2349* but only partial conversion of the substrate by *C. scindens baiH* (Fig. 5d).

### Steroid reductases are prevalent in the human gut microbiome

Given the potential role of hormone-metabolizing enzymes in regulating host hormone balance, we sought to investigate their prevalence and relative abundance across the human population. Considering the known variations in steroid hormone production between males and females and the differential health effects of these hormones, we aimed to determine whether these genes are prevalent in the human gut and whether their presence varies by sex. We gathered 1549 metagenomic samples from 13 studies and conducted a comprehensive analysis to assess the presence and relative abundance of these enzymes across diverse populations (Supplementary Data 3).

**Fig. 6:**
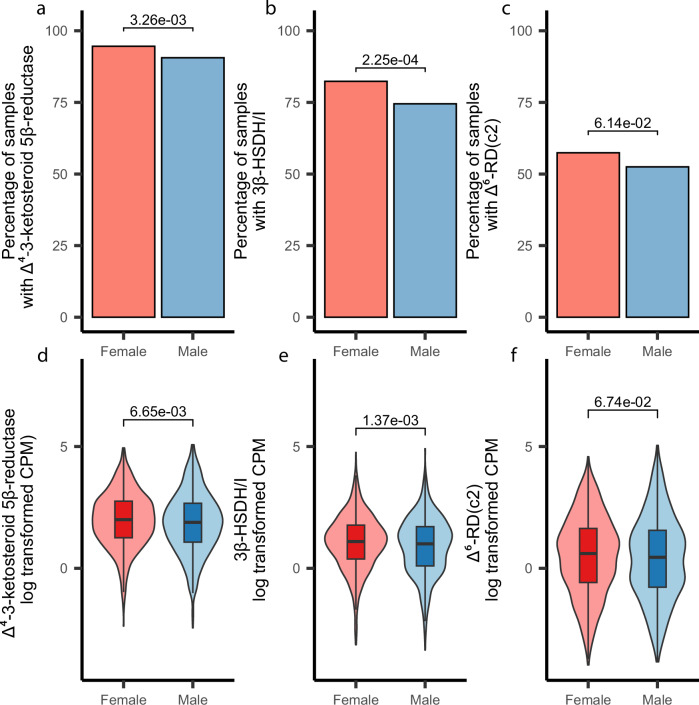
Presence and abundance of steroid hormone reductases in human gut metagenomes. **a–c** Bar plots showing the presence of Δ^4^-3-ketosteroid 5β-reductase (**a**), 3β-HSDH/I (**b**), and Δ^6^-RD(c2) (**c**) in the human gut metagenomes. Samples with greater than one CPM mapping to the gene were considered to have the gene present. Statistical comparisons of gene presence were based on a one-sided two-proportion z-test, testing the hypothesis that gene prevalence is higher in females. **d–f** Plots showing the abundance of Δ^4^-3-ketosteroid 5β-reductase (**d**), 3β-HSDH/I (**e**), and Δ^6^-RD(c2) (**f**) in human gut metagenomes from male and female-derived samples. Plots are shown based on natural log-transformed CPM values (samples with zero counts were excluded from the plot). Statistical comparisons are based on one-sided Wilcoxon rank-sum tests, which test whether the CPM values of female-derived samples are higher than those of male-derived samples. All plots show analyses based on a dataset containing 890 female-derived samples and 659 male-derived samples. Box plots represent the median (center line), with the bounds of the box indicating the 25th and 75th percentiles (interquartile range, IQR). Whiskers extend to the most extreme data points within 1.5×IQR from the lower and upper quartiles.

The presence of Δ^4^-3-ketosteroid 5β-reductase was high, being identified in 94.61% of female samples and 90.59% of male samples, providing evidence that steroid metabolism is a common function in the human gut microbiome (Fig. 6a). Both 3β-HSDH/I and Δ^6^-RD(c2) were less prevalent but were still common, being found in 82.36% and 57.42% of females and 74.51% and 52.50% of male samples, respectively (Fig. 6b). Although the prevalence and relative abundance of Δ^4^-3-ketosteroid 5β-reductase and 3β-HSDH/I were found to be significantly different between sexes, these differences were relatively small. Overall, the widespread detection of these enzymes across human gut metagenomes highlights that microbial steroid metabolism is a core function of the human gut microbiome.

## Discussion

In this study, we identified and characterized three novel families of steroid hormone-metabolizing enzymes found in mammalian gut bacteria: steroid 5β-reductase, 3β-hydroxysteroid dehydrogenase/Δ^5-4^ isomerase, and Δ^6^-3-ketosteroid reductase. In some cases, 5β-reductase and 3β-hydroxysteroid dehydrogenase/Δ^5-4^ isomerase domains are fused within a single gene to form a multi-functional enzyme that catalyzes multiple steps of steroid metabolism. These enzymes mediate the conversion of diverse steroid hormones and synthetic progestins. Genes encoding these enzymes are prevalent in human gut metagenomes across diverse cohorts, suggesting that these microbial activities are common and may contribute significantly to steroid metabolism in the gut environment. By elucidating the genes and enzymatic activities involved in these microbial transformations, our study provides the necessary molecular foundation for future mechanistic investigations.

Despite the potential physiological importance of microbial enzymes in metabolizing host-derived steroid hormones, identifying these enzymes remains challenging due to the immense diversity of the gut microbiota, the difficulty of culturing strict anaerobes, and the lack of genetic tools for manipulating many of these organisms. As shown in early studies, specific Clostridia species can reduce progesterone and related steroids^19,20,24^; however, the genes responsible for these transformations have remained unknown for decades. Here, we leveraged evolutionary information encoded in gene sequences and genomic context to predict function, using phylogenetic reconstruction and comparative genomics as tools to guide enzyme discovery. We identified a novel 3β-hydroxysteroid dehydrogenase/Δ^5-4^ isomerase that is often fused to 5β-reductase in diverse gut-associated taxa. The frequent colocalization and fusion of these enzymes suggests selective pressure to streamline multi-step progesterone transformations. These evolutionary signatures, such as gene fusions, conserved operons, and clade-specific expansions, provide powerful clues into gene function, even in the absence of culturable strains. Functional validation of these enzymes in native gut bacterial strains remains a critical next step, as gene regulation and cellular context may significantly affect enzyme activity. Future studies aimed at isolating and cultivating these bacteria and developing genetic tools for their manipulation will be essential to elucidate the in vivo roles and regulation of these microbial steroid pathways.

Although our original motivation was to identify the enzymes responsible for the known transformations of progesterone and related steroid hormones, our findings revealed that these enzymes may act on a broader range of steroid substrates, such as bile acids and phytosterols. This observation raises the possibility that they participate in a more complex and interconnected network of steroid metabolism^49,50^. While oxidized bile acid intermediates share structural similarities with gonadal steroids and may represent potential substrates, these intermediates are typically transient, produced by a narrow set of *bai*-containing species, and are not known to accumulate broadly in the gut. This complexity is shaped by additional microbial processes, such as sulfonation^51^, and deconjugation, which influence the chemical form and bioavailability of progesterone and its derivatives. Functional redundancy, strain-level variation, and interactions with other microbial taxa further affect how these metabolic activities unfold in vivo. Understanding this intricate system requires more focused efforts to define the specific roles of individual enzymes and microbial species. Identifying the enzymes involved, as we have done here, is an essential first step toward dissecting this dynamic metabolic network and its potential impact on host hormone regulation.

While our biochemical and genomic analyses reveal that gut bacteria encode enzymes capable of transforming pregnenolone, progesterone and related steroids—including producing neuroactive metabolites such as epipregnanolone—the in vivo physiological relevance of these microbial activities remains to be established. Many 3-oxo-Δ⁴ steroids are extensively reduced by the liver into 3α,5α; 3β,5α; 3α,5β; or 3β,5β isomers, a process that inactivates hormone signaling and facilitates excretion. However, studies have shown that a subset of unconjugated and sulfated 3β-hydroxy-5-ene steroids reach the gut in measurable quantities, providing a niche for microbial enzymes to act on substrates not fully processed by the host. A major challenge is that many steroid metabolites in feces originate from both host and microbial sources, making it difficult to disentangle their origins. The host liver performs extensive 5α- and 5β-reductions to inactivate steroids^52^, which may overshadow microbial contributions. Additionally, steroids excreted into bile can undergo further modification by gut microbes through multi-step “ping-pong” interactions involving multiple taxa^53^, complicating attribution. To address this, future studies should quantify substrate concentrations in the gut and measure whether microbially derived products reach the circulation at physiologically relevant levels. Gnotobiotic or antibiotic-treated animal models colonized with defined microbial strains will be essential to assess whether these pathways influence host steroid pools or receptor signaling. Because microbial transformations often occur in the distal gut, it is also important to determine whether these metabolites are absorbed or simply excreted. Finally, subtle microbial effects may be buffered by host endocrine regulation, requiring sensitive metabolomic and isotope tracing approaches. Despite these challenges, our identification of candidate microbial enzymes provides a necessary foundation for future mechanistic studies linking gut microbial steroid metabolism to host endocrine function.

## Methods

### Anaerobic bacteria culturing

Bacterial strains were obtained from the NIH Biodefense and Emerging Infections Research Resources Repository (BEI) (Supplementary Data 4), including *Clostridium innocuum* 6_1_30, *Clostridium paraputrificum* NCTC11833, *Peptoclostridium difficile*, Strain CD160*, Clostridium symbiosum* WAL-14163*, Ruminococcus gnavus* CC55_001C*, Eubacterium ramulus* ATCC29099*, and Escherichia coli*, K-12, Strain IM93. The strains were inoculated from glycerol stocks and incubated under anaerobic conditions (90% N_2_, 5% CO_2_, and 5% H_2_) at 37 °C in an anaerobic chamber (Coy Laboratory Products). The growth medium consisted of 50 mL of liquid brain-heart infusion (BHI) broth (Research Products International, B11000) supplemented with 1 mg per 100 mL of sterile-filtered progesterone or progestin dissolved in ethyl alcohol (EtOH, KOPTEC). Cultures were anaerobically incubated at 37 °C for 24–48 h prior to the introduction of steroid hormones, and then incubated for an additional 48 h before chloroform extraction.

### UHPLC-MS analysis of steroid hormones

After incubation, the bacterial cultures were centrifuged at 3260 × g for 10 min to pellet the cells. The supernatant was then combined with chloroform (4 L; Fisher Chemical, 234522) in a separatory funnel for organic extraction. After separation, the chloroform-steroid hormone solution was air-dried under a fan until the chloroform evaporated completely. Subsequently, 1.5 mL methyl alcohol (4 L; Fisher Chemical, 232699) was added to the dried samples, which were then vortexed to ensure metabolite resuspension. The samples were diluted 1:4 prior to analysis.

The samples were analyzed using a Bruker Maxis-II QTOF ultra-high-resolution Q-TOF mass spectrometer coupled with a Waters Acquity I-Class PLUS LC system. Liquid chromatography separation was performed on a Phenomenex Kinetex C18 100 Å LC Column (100 × 3 mm, 2.6 μm particle size) with mobile phase A (water with 0.1% formic acid) and mobile phase B (acetonitrile with 0.1% formic acid). The gradient program was initially set at 10% B for 2 min. The linear gradient was increased to 90% B for 10 min, followed by maintenance for 4 min, before returning to 10% B for 1 min, and maintained for 3 min. The column was maintained at 4 °C. The injection volume was 5 μl. Mass spectra were acquired under positive electrospray ionization with an ion spray voltage of 4500 V. The source temperature (dry gas) was set at 220 °C at a flow rate of 5.0 L/min. All data were visualized using Bruker Compass Analysis software.

### Hydrophobic compound preparation

All of the sterols used in this study were directly dissolved in ethyl alcohol at a concentration of 1 mg/mL and added to the media. For LC/MS, pregnenolone, progesterone, 5β-dihydroprogesterone, epipregnanolone, 6-dehydroprogesterone, cortisone, hydrocortisone, and all progestins (medroxyprogesterone 17 acetate, levonorgestrel, norgestrel, norgestimate, drospirenone, nomegestrol acetate, norethindrone acetate, and ethynodiol diacetate) were suspended in ethyl alcohol and immediately added to the BHI or Luria-Bertani (LB) broth to undergo reaction. Cholestenone was dissolved in ethyl alcohol and shaken at 220 RPM and 25 °C for 18 h before being added to the medium.

### Identifying candidate Δ^4^-3-ketosteroid 5β-reductases

Three genomes from the experimentally confirmed progesterone reducers, *C. innocuum* 6_1_30 (GCA_000183585.2), *C. paraputrificum* NCTC11833 (GCA_900447045.1), and *R. gnavus* CC55_001C (GCA_000507805.1), and three genomes from the closely related non-progesterone reducers, *C. difficile* ATCC 9689 (GCA_001077535.1), *C. symbiosum* WAL-14163 (GCA_000189595.1), and *E. ramulus* ATCC 29099 (GCA_000469345.1), were downloaded from the NCBI database. The genomes were annotated using Prokka (v1.14.5). OrthoFinder (v2.5.5) was used to group the predicted protein sequences into orthogroups using an e-value cutoff of 1e-120. The taxonomic distribution of each orthogroup was profiled to identify the orthogroups present in progesterone-reducing species and those absent in non-progesterone-reducing species. Only 13 of the OGs shared the same taxonomic distribution. Of these OGs, only two, *ci2350* and *ci0705*, were annotated as Old Yellow Enzymes and further investigated as putative Δ^4^-3-ketosteroid 5β-reductase.

### Cloning and transformation of expression constructs

Genes of interest, including *3β-HSDH* from *C. innocuum*, *3β-HSDH* and *3α-HSDH* from *R. gnavus* CC55_001C, *baiCD* and *baiH* from *C. scindens*, and *ci2349* and *ci2350*, were either PCR-amplified from genomic DNA or synthesized (GeneScript, Piscataway, NJ, USA). PCR amplification was performed using OneTaq Master Mix (NEB, M0482) with primers designed in SnapGene and ordered from GeneWiz (Supplementary Data 5).

Plasmid backbones used included pCW-lic (Addgene 26908), pET28a(+) (Addgene 69864-3), and pET11. Vectors were digested with the appropriate restriction enzymes (NdeI/HindIII-HF, NdeI/XhoI, or NdeI/BamHI), and inserts were assembled via Gibson assembly (NEB, E2611S). Construct-specific cloning strategies were selected based on the expression needs and compatibility with downstream applications.

Following assembly, the constructs were transformed into chemically competent *E. coli* strains according to the manufacturer’s instructions: NEB 10-beta (NEB, C3019) for pCW-lic plasmids and NEB T7 Express lysY/Iq (NEB, C3013I) for pET28a(+) and pET11 vectors. Transformed cells were plated on Luria-Bertani (LB) agar containing the appropriate antibiotics: 100 μg/mL carbenicillin for pCW-lic and pET11 constructs or 50 μg/mL kanamycin for pET28a(+) constructs. Colonies harboring recombinant plasmids were verified using Oxford Nanopore sequencing (Plasmidsaurus).

### Transformed *E. coli* culturing

Transformed *E. coli* strains were cultured by inoculating glycerol stocks derived from agar colony plates into 250 mL of LB medium (Sigma-Aldrich, 0000324647) supplemented with either 50 μg/mL kanamycin or 100 μg/mL carbenicillin (Chem-Impex, 001453-230228 & Bio Basic, Q3030310). The cultures were shaken aerobically at 37 °C for 20 h. Subsequently, the bacterial cells were harvested by centrifugation at 3260 × g for 10 min and transferred to an anaerobic chamber. The cell pellet was resuspended in 250 mL of LB medium containing 50 μg/mL kanamycin or 100 μg/mL carbenicillin and 0.4 mM IPTG to induce the expression of the protein. The strains were incubated anaerobically at 37 °C for 48 h prior to chloroform extraction. All incubations were performed using uniform steroid concentrations (1 mg per 100 mL) and identical incubation durations to ensure comparability across assays.

### Protein purification

*E. coli* strains transformed with *ci2350* were grown aerobically in LB medium containing 50 µg/mL kanamycin overnight at 37°C. A 5 mL overnight culture was used to inoculate 500 mL of fresh LB, and cells were grown to an OD of ~0.5. Protein expression was induced with 0.4 mM IPTG, and the cultures were incubated overnight at 25°C. The cells were harvested by centrifugation at 3260 × g, and the supernatant was discarded. Pellets were stored at –20°C, then thawed on ice, and resuspended in lysis buffer (0.1 M sodium phosphate, 0.2 M NaCl, 20 mM BME, pH 6.5) supplemented with lysozyme (Sigma-Aldrich, L6876-1G), benzonase (Sigma-Aldrich, E8263-5KU), and protease inhibitor (Sigma-Aldrich, 04693124001). Cells were lysed by sonication at 4°C using a Branson 550 sonicator at 40% amplitude, 10 s on/off pulses for a total of 2 min. Lysates were clarified by centrifugation at 14,000 × g for 10 min, and the supernatant was filtered through a 0.22 µm PES syringe filter (Celltreat, 229746). The filtered lysate was loaded onto an Ni²⁺ HisTrap Excel column (Cytiva, 17371205) using a peristaltic pump. The column was washed with a buffer containing 20 mM imidazole to remove non-specifically bound proteins, and the target protein was eluted with a buffer containing 200 mM imidazole. Eluted protein was concentrated and buffer-exchanged into 0.1 M sodium phosphate, 0.2 M NaCl, and 20 mM BME (pH 6.5) using Amicon Ultra centrifugal filters (30 kDa MWCO, Millipore Sigma, UFC5030). Expression of recombinant ci2350 was confirmed by SDS-PAGE analysis prior to purification.

### Isothermal titration calorimetry

Protein stocks were concentrated to the desired concentrations in 0.1 M sodium phosphate, 0.2 M sodium chloride, and 20 mM BME (pH 6.5). All compounds tested in the ITC assays were prepared in the same buffer. Owing to their hydrophobic nature, most ligands required additional preparation to ensure solubility: compounds were suspended in phosphate buffer at 10 mM concentration in vials and shaken at room temperature for 24 h before being diluted to working concentrations with the addition of 0.5 mM CHAPS (AG Scientific, C-1019). Equal concentrations of CHAPS were then added to each protein sample. All the protein and ligand solutions were degassed prior to use.

ITC experiments were performed at 25°C using a TA Instruments Nano ITC instrument. Progesterone (Sigma-Aldrich, P8783-1G), 6-dehydroprogesterone (Toronto Research Chemical, D231520), cortisone (Cayman Chemical, 20739), and hydrocortisone (Cayman Chemical, 20763) were assayed using standard titrations with the ligand in the syringe and the protein in the cell. The ligand was injected into 3.1 µL aliquots at 180-second intervals with a stirring speed of 150 RPM. Heats of dilution were subtracted, and the data were analyzed using NanoAnalyze software (TA Instruments, v3.7.0). Binding curves were generated to calculate the enthalpy change (ΔH), entropy change (ΔS), stoichiometry (n), and dissociation constant (Kd) based on the best-fit binding models. Independent binding models were required for progesterone, 6-dehydroprogesterone, cortisone, and hydrocortisone. Protein and ligand concentrations are explained in the figure captions.

### Phylogenetic tree construction

Representative genomes were downloaded from the Genome Taxonomy Database (GTDB, version r214)^54^ and annotated using Prokka (version 1.14.6)^55^ to identify protein sequences. Sequences assigned to COG1092 were identified using eggNOG-Mapper (version 2.1.3)^56^. Comparative analysis included a BLASTp search (version 2.15.0 + )^57^ using the query sequence ci2350/ci2349 against the identified sequences, setting a limit for the top 500 hits. Sequence alignment was performed using the MUSCLE software (version 5.1)^58^. The alignments were subsequently trimmed to retain only those regions mapped to the ‘Old Yellow Enzyme’ domain. Additionally, areas exhibiting more than 95% gaps were removed to enhance alignment quality. Sequences shorter than 500 amino acids for ci2350 homologs and 450 amino acids for ci2349 homologs were excluded using SeqKit (version 2.7.0)^59^ to ensure the robustness of the analysis. Phylogenetic analysis was performed using IQ-TREE (version 2.1.2)^60^, employing the LG + I + G4 model to handle rate heterogeneities across sites. The reliability of the phylogenetic trees was evaluated using 1000 ultrafast bootstrap replicates. The trees were visualized using an Interactive Tree Of Life (iTOL)^61^.

### Structural prediction and identifying catalytic residues

The structures of the putative 5β-reductase protein *Clostridium innocuum* 6_1_30 (GCA_000183585.2) were predicted using AlphaFold (version 2.2.0)^62^. Binding pockets were predicted using fpocket (v4.0) with the default parameters. The pockets were compared to homologous *E. coli* 2,4-Dienoyl CoA Reductase (PDB: 1PS9) to identify the putative substrate-binding regions and catalytic residues. The structure of progesterone (PubChem compound identifier: 5994) was docked onto the predicted structure of 5β-reductase using AutoDock Vina (v1.2.5)^63^. The docking simulation was performed within 15 Å × 15 Å × 15 Å cubes centered on the center points of the chosen fpocket^64^ substrate-binding pocket with an exhaustiveness set to 32. Docking results were visualized using PyMOL software. The 5β-reductase (ci2350) active site residues were identified using TMalign^65^ on BaiCD and *E. coli* 2,4- Dienoyl CoA Reductase (PDB:1PS9). Based on the current literature, ci2350 residues that aligned with the known active site residues in 1PS9 were further investigated.

### Profiling of steroid hormone reductases in metagenomic samples

HMM profiles were built for 5β-reductase, Δ^6^-RD(c2), and 3β-HSDH/I enzymes, based on the identified clades for each gene. First, the protein sequences for each gene were aligned using ClustalO (version 1.2.4)^66^ and HMM profiles were constructed for each gene set using the *hmmbuild* tool from HMMER (version 3.4)^67^(profiles for each gene are available as part of the ProkFunFind software package available (https://github.com/nlm-irp-jianglab/ProkFunFind). For the fused Δ^4^-3-ketosteroid 5β-reductase and 3β-HSDH/I, the regions of the sequence corresponding to each fused domain were extracted and separately included in the Δ^4^-3-ketosteroid 5β-reductase or 3β-HSDH/I datasets. The HMMER *hmmsearch* tool (version 3.4)^67^ was used to search for putative hits to each enzyme in the collection of all genomes in GTDB, with the hits being filtered by E-values of 1×10^-240^, 1×10^-220^, and 1×10^-260^ for the 5β-reductase, 3β-HSDH/I, and Δ^6^-RD(c2) searches, respectively. The nucleotide sequences for the respective hits were then extracted, with fused Δ^4^-3-ketosteroid 5β-reductase and 3β-HSDH/I genes split into separate parts, and reads were mapped to them in the subsequent metagenomic analysis.

A collection of 890 female-and 659 male-derived gut metagenomic samples from 13 studies was used to analyze the presence of steroid metabolism genes (Supplementary Data 3). Each metagenome was downloaded from SRA, and adapter sequences were trimmed from the reads using Trim-Galore with default settings^68^. The reads were then mapped to the human reference genome (assembly T2T-CHM13v2.0), and potential human contaminant reads were removed using Samtools (version 1.16.1)^69^. The quality-trimmed reads were aligned to the gene reference sets using Bowtie2 (version 2.5.3)^70^. For the biosamples associated with multiple runs, the mapping counts for each gene were summed across all runs for each sample. Only samples with at least one million reads combined with quality-trimmed reads per biosample were further analyzed. Read mapping was then normalized to a count per million (CPM) value for each gene by dividing the number of mapped reads by the total reads and multiplying the value by one million. If a gene had a CPM greater than 1 in a sample, it was considered to be present in that sample. The proportion of samples with each gene present was compared between male- and female-derived samples using a one-sided two-proportion z-test in R (*prop.test*). The CPM values between groups were compared using one-sided Wilcoxon rank-sum tests in R.

### Statistics and reproducibility

No statistical method was used to predetermine sample size. No data were excluded from the analysis. The experiments were not randomized, and investigators were not blinded to allocation during experiments and outcome assessment.

## Supplementary information

Supplementary Information

Description of Additional Supplementary Files

Supplementary Data 1

Supplementary Data 2

Supplementary Data 3

Supplementary Data 4

Supplementary Data 5

Transparent Peer Review file

## Data Availability

Gene information related to the identified Δ^4-3^-ketosteroid 5β-reductases and Δ^6^-3-ketosteroid reductases is available in Supplementary Data 1 and 2, respectively. Publicly available metagenomic data analyzed in this study is available in Supplementary Data 3. Bacterial strains used in this study, along with their genomes, are available in Supplementary Data 4. Primers used for cloning gene sequences are provided in Supplementary Data 5. All genomic data analyzed in this study are available through the GTDB. The SRR accession numbers of all metagenomic samples are listed in the Supplementary Data and are available on Sequence Read Archive (SRA) at NCBI. Source data for Fig. 6 are provided with this paper.

## Code availability

Code required to replicate the read mapping analysis is available on GitHub (github.com/nlm-irp-jianglab/steroid-reductase-bioinfo/tree/main) (10.5281/zenodo.15675720). Hidden Markov model profiles for the identification of steroid hormone reductases were also provided as part of the ProkFunFind search tool (https://github.com/nlm-irp-jianglab/ProkFunFind/tree/master/data/Steroid_reductase).
