# Supplementary material for: Identification of gut bacteria reductases that biotransform steroid hormones
Source: Nat Commun. 2025 Jul 8;16:6285. doi: 10.1038/s41467-025-61425-6 (PMC12238238; doi:10.1038/s41467-025-61425-6)
Supplement: Supplementary file 1 — Supplementary Information [file 41467_2025_61425_MOESM1_ESM.pdf]

Identification of gut bacteria reductases that biotransform steroid hormones

Authors:

Gabriela Arp[1], Angela K Jiang[1,2], Keith Dufault-Thompson[2], Sophia Levy[1], Aoshu Zhong[3], Jyotsna Talreja Wassan[2,4], Maggie R Grant[1], Yue Li[5], Brantley Hall[1,6*], Xiaofang Jiang[2,*]

Affiliations:

[1]Department of Cell Biology and Molecular Genetics, University of Maryland, College Park, College Park, Maryland, USA

[2]National Library of Medicine, National Institutes of Health, Bethesda, Maryland, USA

[3]Division of Molecular and Cellular Biology, Eunice Kennedy Shriver National Institute of Child Health and Human Development, National Institutes of Health, Bethesda, Maryland, USA

[4]Department of Computer Science, Maitreyi College University of Delhi, Delhi, India

[5]Department of Chemistry and Biochemistry, University of Maryland, College Park, College Park, Maryland, USA

[6]Center for Bioinformatics and Computational Biology, University of Maryland, College Park, College Park, Maryland, USA

[*]Corresponding authors: xiaofang.jiang@nih.gov, brantley@umd.edu

Supplementary Figures

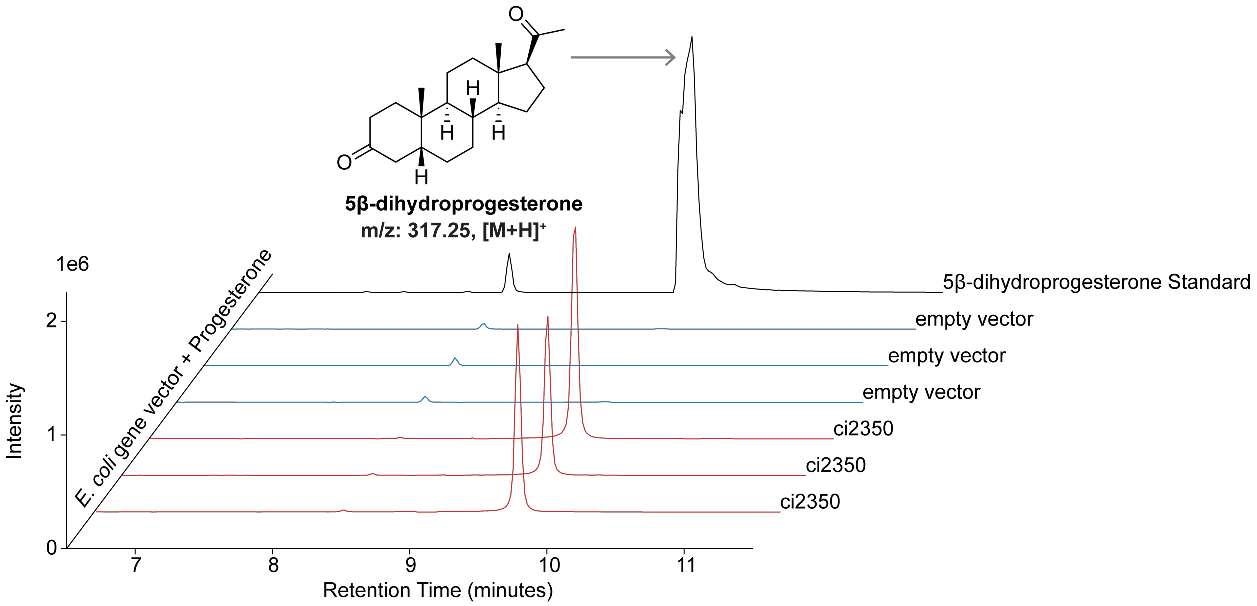

Supplementary Fig. 1: LC-MS analysis of progesterone-to-5β-dihydroprogesterone biotransformation by *Escherichia coli* heterologously expressing *ci2350*. Extracted ion chromatograms (EICs) of 5β-dihydroprogesterone in *E. coli* carrying either an empty vector (blue traces) or a plasmid encoding the gene *ci2350* (red traces).

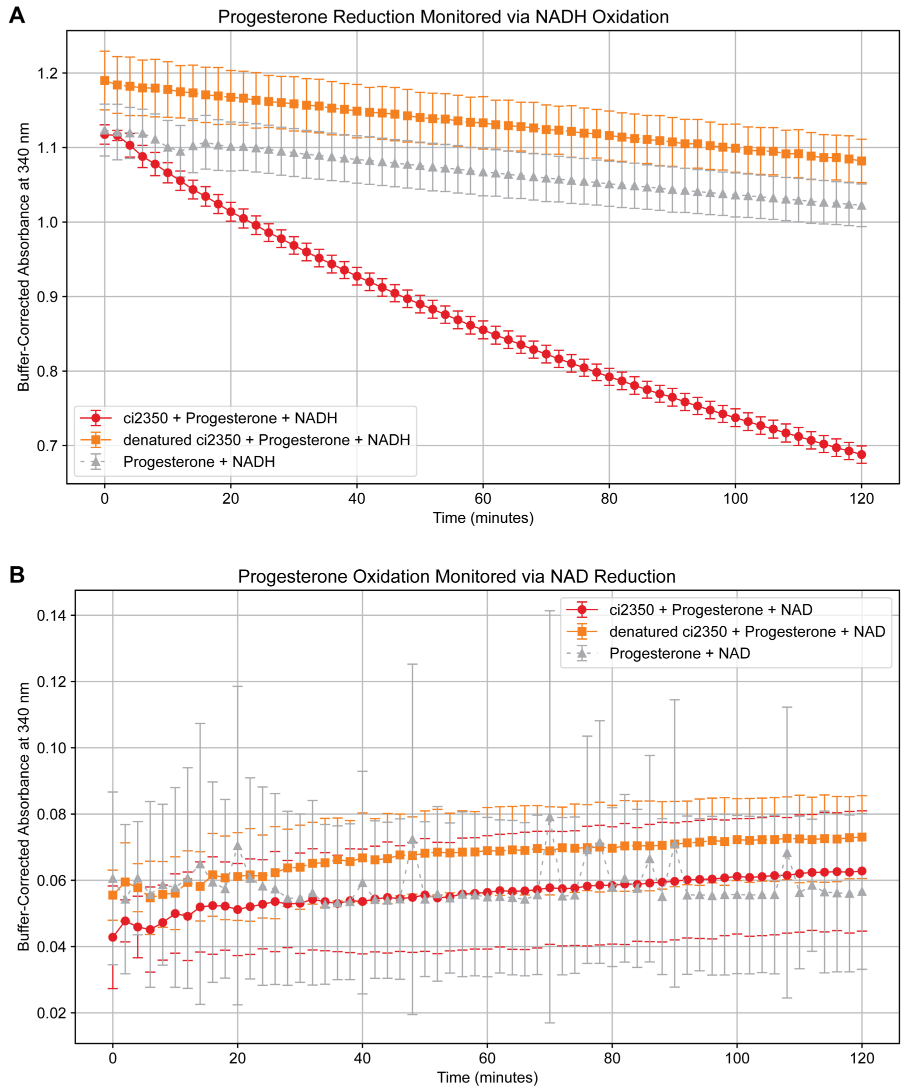

Supplementary Fig. 2: ***Progesterone biotransformation by ci2350 monitored via NADH oxidation and NAD⁺ reduction.*** (A) **Progesterone reduction by ci2350 coupled with NADH.** The activity of purified recombinant ci2350 was monitored by measuring the decrease in absorbance at 340 nm, corresponding to NADH oxidation, over 120 minutes. Reactions contained progesterone and NADH with active ci2350 (red circles), heat-denatured ci2350 (orange squares), or no enzyme (gray triangles). Only the active enzyme condition showed a sustained decrease in NADH absorbance, indicating that enzymatic reduction of progesterone is coupled to NADH oxidation. (B) **Progesterone oxidation by ci2350 coupled with NAD^+.** The same enzyme was tested for progesterone oxidation activity by monitoring NADH formation over time. Reactions contained progesterone and NAD^+ with active ci2350 (red circles), heat-denatured ci2350 (orange squares), or no enzyme (gray triangles). All conditions showed minimal change in absorbance, and no sustained increase in NADH signal was observed, suggesting that ci2350 does not catalyze the oxidation of progesterone in the presence of NAD^+. Values represent the mean ± standard deviation of biological replicates (n = 3).

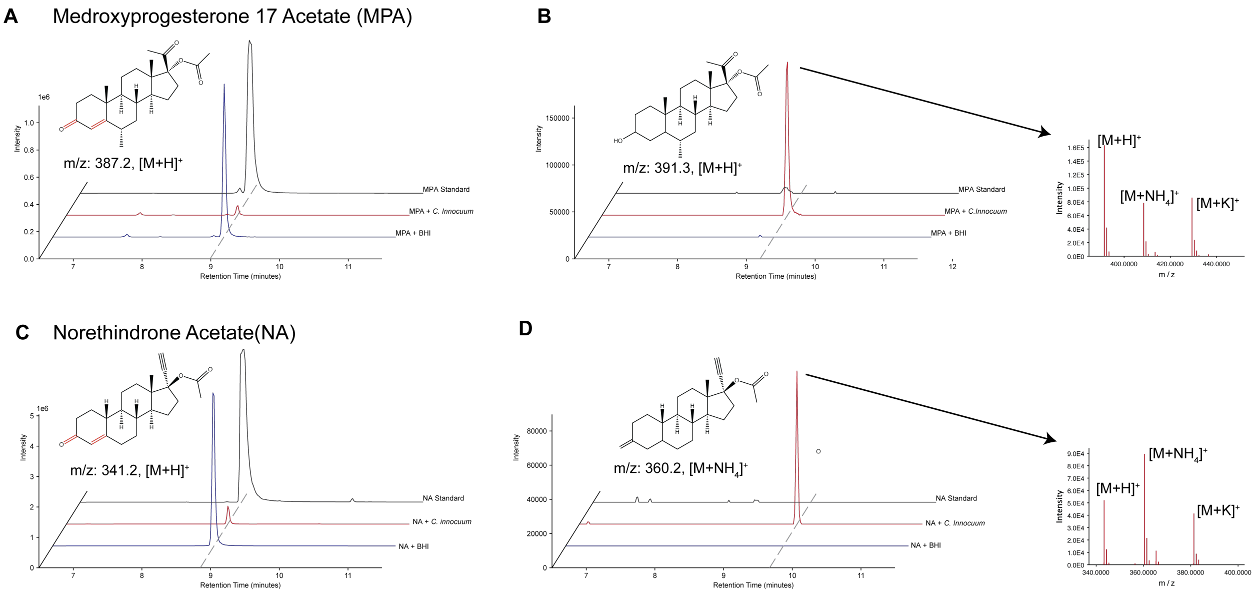

Supplementary Fig. 3: LC-MS analysis of progestins and their reduced products by *C. innocuum*. (A, C) Extracted ion chromatograms (EICs) of medroxyprogesterone acetate (MPA; A) and norethindrone acetate (NA; C) and their biotransformation products after incubation with *C. innocuum*. EICs are shown for authentic standards (black), sterile medium with substrate (blue), and cultures of *C. innocuum* with substrate (red). The diagonal dashed lines indicate the retention times of the reduced metabolites.(B, D) MS^1 spectra of the corresponding metabolite products from MPA (B) and NA (D), confirming that the molecular ion peaks were consistent with the products. The structures of the parent compounds and the proposed reduced metabolites are shown.

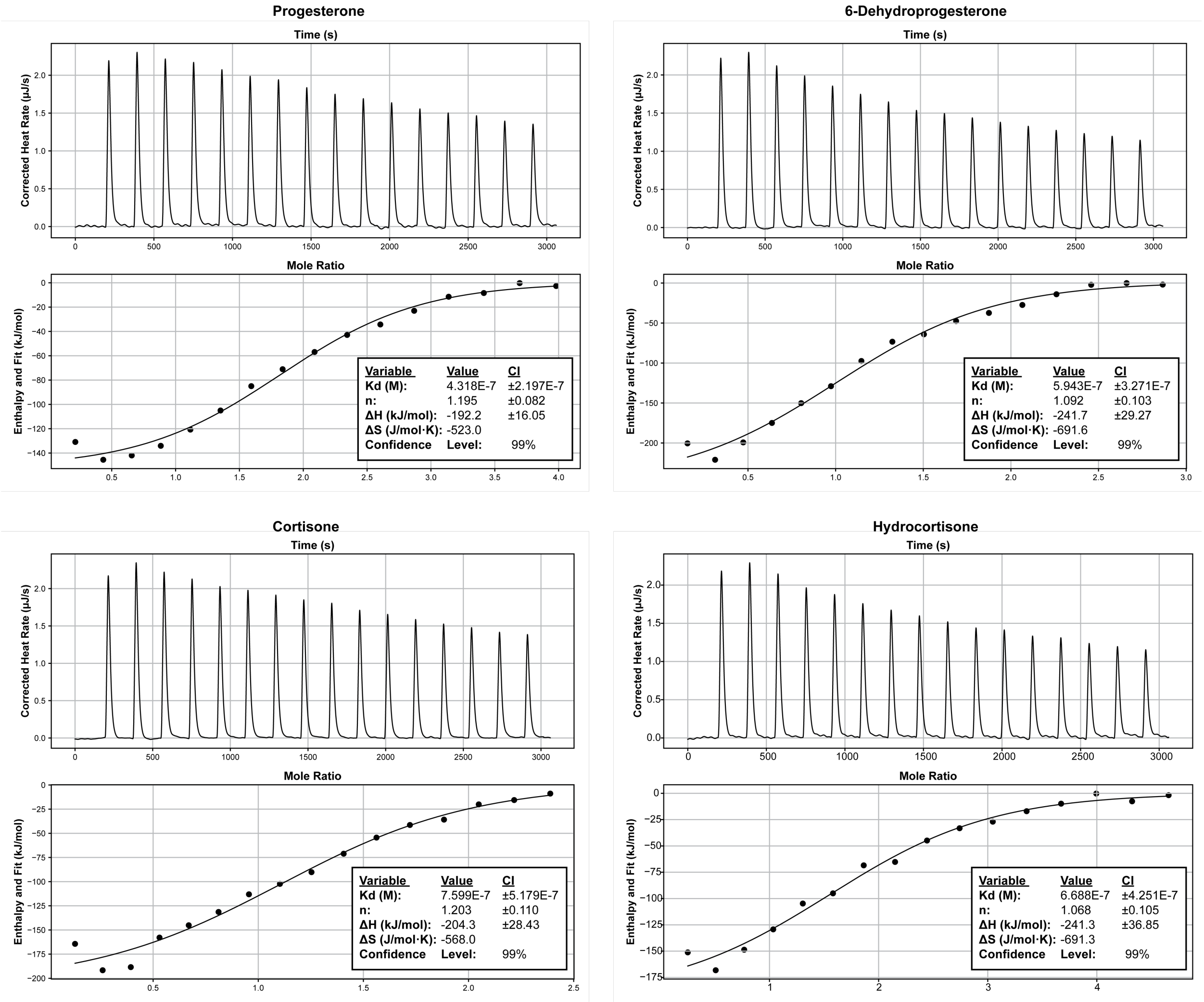

Supplementary Fig. 4: Isothermal titration calorimetry (ITC) analysis of steroid binding to ci2350. Raw data (heats of binding) are shown in the upper graph, and the integrated binding isotherms fitted with an independent binding model are shown in the lower panel. For each titration, the ligand was present within the syringe and successive 3.1µL injections were made into a cell containing the recombinant ci2350 at 25°C. All binding was performed using a 50 µm ligand and 5 µm protein. Thermodynamic parameters were determined, including the binding affinity (Kd), stoichiometry (n), enthalpy change (ΔH), and entropy change (ΔS), all reported with 99% confidence intervals. All ligands exhibited nanomolar affinity and strong exothermic binding, indicating tight and specific interactions with ci2350.

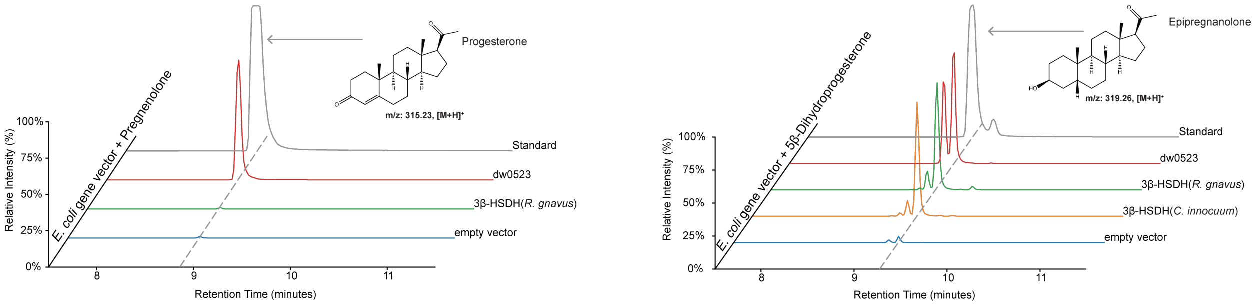

Supplementary Fig. 5: Pregnenolone and 5β-dihydroprogesterone transformation by 3β-HSDH/I and 3β-HSDH transformed *E. coli*. The left panel shows the ion chromatogram for progesterone (m/z 315.23), while the right panel shows the ion chromatograms for the transformation products 5β-dihydroprogesterone (m/z 317.24) and epipregnanolone (m/z 319.28). Each line in the chromatograms represents data from *E. coli* transformed with different genes. The structural formulae of the steroids are displayed adjacent to their corresponding peaks, linking the observed chemical transformations with molecular changes.

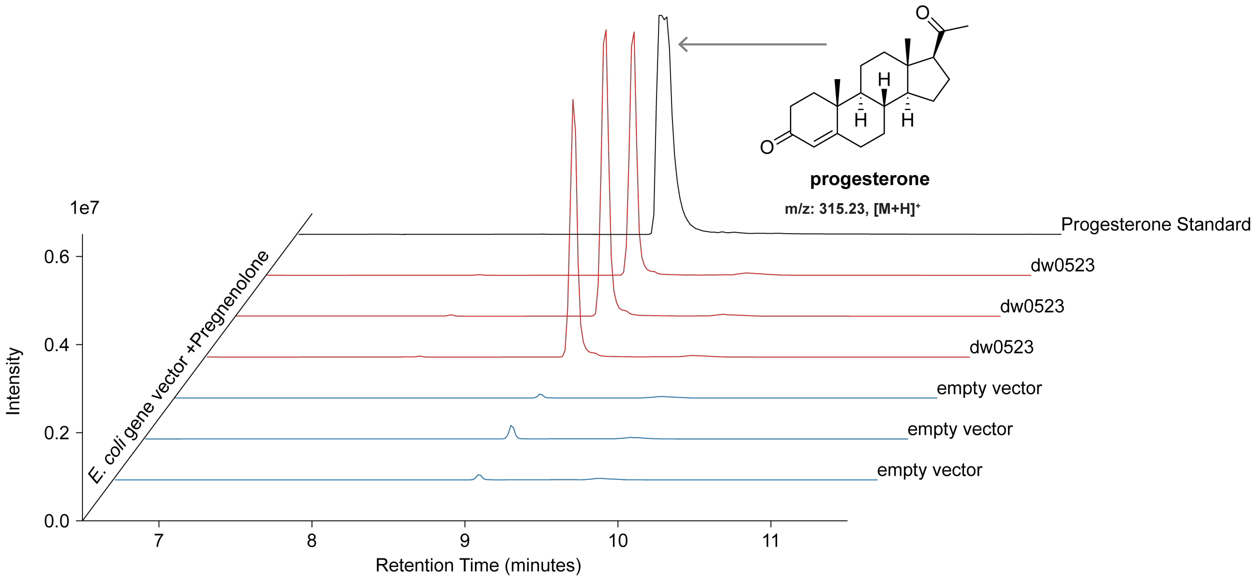

Supplementary Fig. 6: LC-MS analysis of pregnenolone-to-progesterone biotransformation by *E. coli* heterologously expressing *dw0523*. Extracted ion chromatograms (EICs) of pregnenolone from *E. coli* cultures carrying either an empty vector (blue traces) or plasmid encoding *dw0523* (red traces).

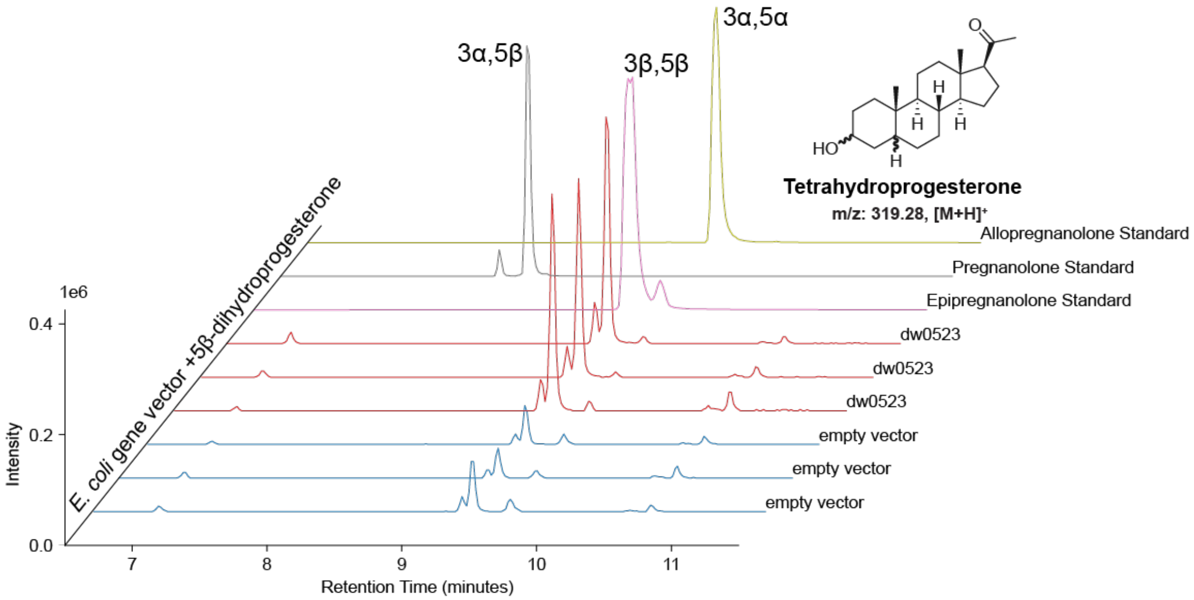

Supplementary Fig. 7: LC-MS analysis of 5β-dihydroprogesterone-to-epipregnanolone biotransformation in *E. coli* heterologously expressing *dw0523*. Extracted ion chromatograms (EICs) of pregnenolone from *E. coli* cultures carrying either an empty vector (blue traces) or a plasmid encoding *dw0523* (red traces).

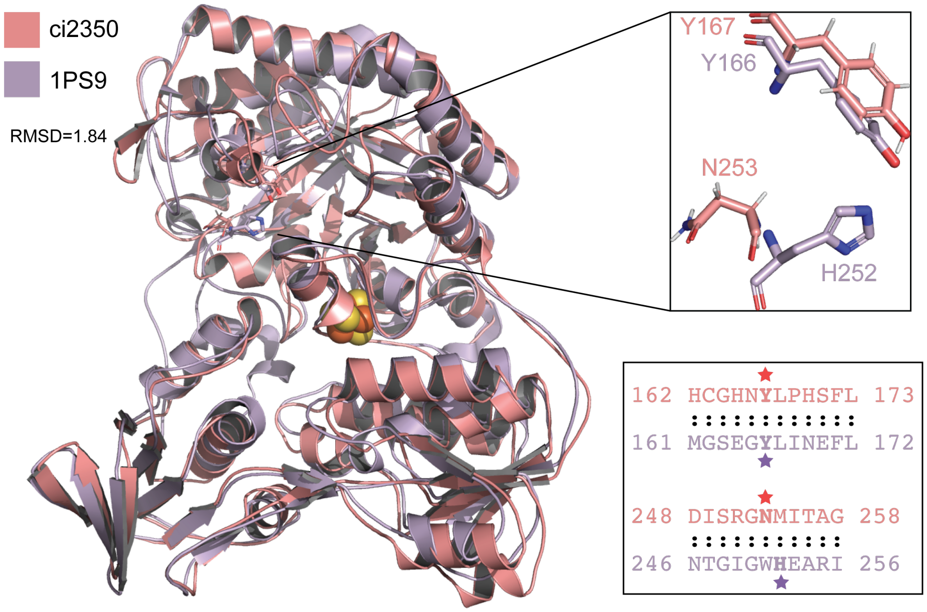

Supplementary Fig. 8: Identification of putative active-site residues in the ci2350 predicted structure. Alignment of the AlphaFold predicted the structure of the ci2350 steroid hormone 5β-reductase enzyme (red) and the structure of the *E. coli* 2,4-dienoyl CoA reductase enzyme (purple) (PDB: 1PS9). The insert on the top right shows the alignment of the putative ci2350 active-site residues with the characterized catalytic residues in the 1PS9 structure. The insert on the bottom right shows subsets of the multiple sequence alignment between the ci2350 and 1PS9 amino acid sequences, with stars indicating the positions of the residues of interest in both protein sequences.

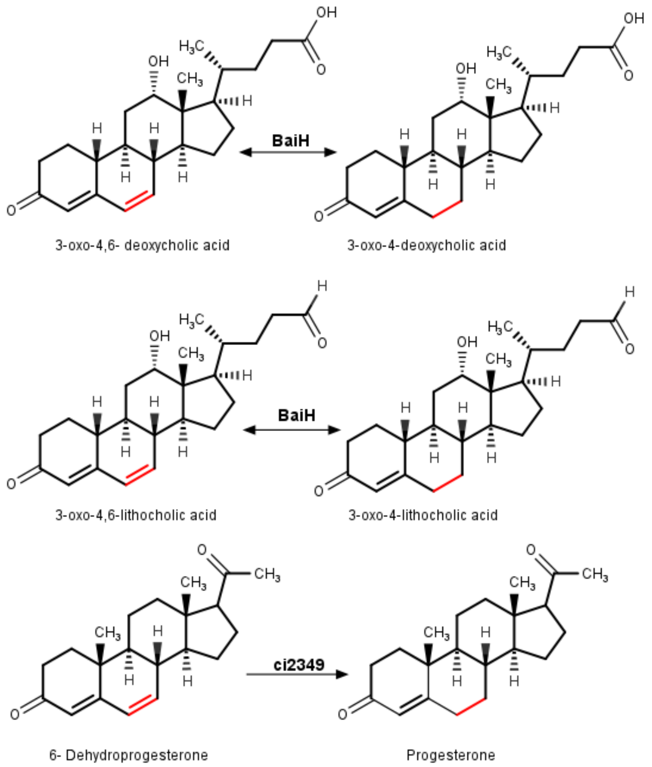

Supplementary Fig. 9: Reactions catalyzed by Δ⁶-3-ketosteroid reductases. Reaction schematics are shown for the reduction reactions catalyzed by BaiH enzymes (top two reactions) and steroid hormone Δ⁶-3-ketosteroid reductases represented by *ci2349* (bottom reaction). The carbon double bonds involved in the reaction are highlighted in red on the left side of the reactions, and the corresponding reduced bonds are highlighted in red on the right side of the reactions.
