# Supplementary material for: Identification of gut bacteria reductases that biotransform steroid hormones
Source: Nat Commun. 2025 Jul 8;16:6285. doi: 10.1038/s41467-025-61425-6 (PMC12238238; doi:10.1038/s41467-025-61425-6)
Supplement: Supplementary file 2 — Description of Additional Supplementary Files [file 41467_2025_61425_MOESM2_ESM.pdf]

File Name: Supplementary Data 1

Description: Supplementary Data 1 contains information related to the proteins present in the Δ4-3-ketosteroid 5β-reductase family. The predicted protein sequence, length, substrate specificity, and taxonomy were determined for each sequence.

File Name: Supplementary Data 2

Description: Supplementary Data 2 contains Δ6-3-ketosteroid reductase identified information related to proteins present in the Δ6-3-ketosteroid reductase family. The predicted protein sequence, protein length, predicted substrate specificity, and taxonomy are provided for each sequence.

File Name: Supplementary Data 3

Description: Supplementary Data 3 contains metagenomic sample information. Metagenomic data analyzed in this study. The table includes the SRA run, sample ids, and associated study information, along with read mapping counts and associated sample biological sex metadata.

File Name: Supplementary Data 4

Description: Supplementary Data 4 contains the strain list. Information about the individual strains used during cloning: The list includes the strain name, BEI strain designation number, and media in which it was grown.

File Name: Supplementary Data 5

Description: Supplementary Data 5 contains primer sequences used for cloning. Primers used for gene amplification. This included the strain, gene name, vector backbone, forward primer sequence, and reverse primer sequence. The capitalized bases in Gibson primers are regions that anneal to the target sequence for amplification. The lowercase bases represent the additional bases included in the primers to create the necessary overlap with the vector backbone for the Gibson assembly reaction.
